# Follicular development and the expression of BAX and vascular endothelial growth factor in transplanted ovaries in uni- and bilateral ovariectomized mice: An experimental study

**DOI:** 10.18502/ijrm.v19i4.9062

**Published:** 2021-04-22

**Authors:** Maryam Dehghan, Shirin Shahbazi, Mojdeh Salehnia

**Affiliations:** ^1^Anatomy Department, Faculty of Medical Sciences, Tarbiat Modares University, Tehran, Iran.; ^2^Medical Genetic Department, Faculty of Medical Sciences, Tarbiat Modares University, Tehran, Iran.

**Keywords:** Autotransplantation, BAX protein, Vascular endothelial growth factor, Ovariectomy, Mice.

## Abstract

**Background:**

Several conflicting results have been reported on the survival and function of transplanted ovaries.

**Objective:**

Evaluation of the follicular development and the expression of vascular endothelial growth factor (VEGF) and Bcl-2-associated X protein (BAX) in ovaries transplanted into uni- and bilaterally ovariectomized mice.

**Materials and Methods:**

In this experimental study, 40 female NMRI mice (21-days-old, 12-15 gr) were ovariectomized uni- and bilaterally (n = 20/ group), while the 8-wk-old mice were considered as intact control group (n = 6). 5 weeks after transplantation at the proestrus stage, the morphology of recovered transplanted ovaries and the proportion of follicles were studied at different developmental stages. The apoptosis cell death by pro-apoptotic protein BAX and the expression of VEGF were evaluated using immunohistochemistry.

**Results:**

In the bilaterally ovariectomized mice, among the 455 counted normal follicles, a lower rate of primordial and primary follicles and a higher rate of preantral and antral follicles were observed (p = 0.002). However, the percentages of preantral and antral follicles, and the corpus luteum were significantly lower in the intact control group (among the 508 counted normal follicles in this group) compared to other transplanted groups (p = 0.002). The number of BAX-positive cells in all groups was not significantly different. The VEGF expression was prominent in vessels of the corpus luteum, and also in the theca layer of large follicles of studied groups.

**Conclusion:**

Early discharge of ovarian reserve was prominent in the bilaterally ovariectomized group but the incidence of apoptotic cells and VEGF expression as angiogenic factor did not differ in both ovariectomized mice. Thus, unilaterally ovariectomy has less side effects on the ovarian reserve compared to bilateral ovariectomy.

## 1. Introduction

Ovarian transplantation is used to restore fertility potential and ovarian function in women with premature ovarian failure and in patients with cancer, and it can be performed using fresh or cryopreserved tissue in an autograft, allograft, and xenograft manner at orthotropic or heterotopic sites (1-4).

Ischemia and degeneration of follicles are important factors, which should be considered during the transplantation of ovarian tissue and it can cause a decline in ovarian reserve and fertility potential (5-8). The vascular condition of the graft site plays a critical role in the reduction of ischemia. Therefore, angiogenesis in transplanted tissue and growth of the ovarian follicles depends on the graft site (5, 6, 8). Vascular endothelial growth factor (VEGF) plays an important role in the posttransplantation angiogenesis of ovarian tissue (9-11). Zand-Vakili and colleagues demonstrated that VEGF, by the formation of new vessels, can enhance the follicular and corpus luteum growth and also their development (11). The kidney capsule has a rich vascular supply and receives about 20% of the cardiac output; thus, it could be considered as an ideal site for ovarian tissue transplantation (12). The uni- and bilaterally ovariectomized mice were used as recipients for ovarian transplantation (13-16). There are several conflicting results regarding the influence of ovariectomy of the recipient animal on the survival and function of transplanted ovarian tissue (12-16). While several studies have reported that the grafted ovarian tissues have been functional in bilaterally ovariectomized recipients (13, 14), others demonstrated that the follicular growth declined in grafted ovarian tissue in unilaterally ovariectomized recipients (15, 16).

Apoptosis cell death plays a fundamental role in follicular atresia. It takes place by two intrinsic and extrinsic pathways, and several pro- and antiapoptotic proteins involve in these pathways (17). BAX as a proapoptotic protein is expressed in both granulosa cells and oocytes. It is an important regulator of follicular growth and atresia, which plays a central role in ovarian cell death by the intrinsic pathway of apoptosis (17, 18). A higher incidence of apoptosis was shown within grafted ovarian tissue (19-22); however, a well-preserved ovarian follicles with a low proportion of cell death in grafted tissue was demonstrated by others (23).

Before usage of any ovariectomized animal models as tissue recipient it is necessary to determine the impact of ovarian removal procedure on the incidence of apoptosis and angiogenesis of transplanted ovarian tissue. Up to the best of our knowledge, there is no attention paid to compare the incidence of apoptosis in grafted ovaries in uni- and bilaterally ovariectomized samples. Thus, this study was aimed to evaluate and compare the rate of follicular development, the percentages of apoptosis cell death using immunohistochemistry for BAX, and the expression of VEFG at the protein level in auto-transplanted ovaries into a uni and bilaterally ovariectomized recipient.

## 2. Materials and Methods

### Chemicals

All chemicals were obtained from Sigma-Aldrich (Germany) except if mentioned otherwise.

### Animals

In this experimental study, forty 21-days-old female NMRI mice (n = 40) were kept under a controlled condition (20-24°C, 12-hr light/dark cycles and 40-50% humidity) in the animal house of Tarbiat Modares University. Additionally, 8 wk old female mice (n = 6) used for the collection of ovaries for immunohistochemistry as a tissue control group.

### Experimental design 

The animals in experimental groups were divided into three groups as follows:

Group A (unilaterally ovariectomized mice): The right ovary of the mouse was removed and transplanted into kidney capsule while the left ovary was kept intact (n = 20).

Group B: (bilaterally ovariectomized mice): Both ovaries of the animals were removed and the right ovary was inserted under the right kidney capsule and the left ovary was withdrawn (n = 20).

Group C: The mice were 8-wk old and their both ovaries kept intact (n = 6).

Five wk after the transplantation, the morphology of grafted ovaries was studied using hematoxylin and eosin (H&E) staining. Also, the apoptosis cell death and angiogenesis were evaluated by immunohistochemistry staining using anti-BAX and VEGF antibodies.

### Ovarian removal and autotransplantation

Autotransplantation procedure was done according to a previous work (24). Animals were anesthetized with i.p. injection of ketamine (50 mg/kg) and xylazine (5 mg/kg).

(i) In group A (n = 20 in 10 repeats): Through a dorsal horizontal incision, the right ovary of mice was removed and put into the right kidney capsule while the left ovary was kept intact.

(ii) In group B (n = 20 in 10 repeats): Through a dorsal horizontal incision, both ovaries of mice were removed and the right ovary was inserted under the right kidney capsule and the left ovary was withdrawn.

Finally, after the transplantation, their body walls and skin incisions were closed. All procedures were performed under aseptic conditions. Five weeks after the transplantation at the proestrus phase, the mice in all groups were sacrificed by cervical dislocation and their transplanted ovaries were recovered and collected for the following assessments. Also, both ovaries of 8-wk old female mice were collected at the proestrus phase and considered as the intact control group (n = 6 mice in six repeats).

### Vaginal cytology 

Five wk after the transplantation, the stages of mice estrous cycle at proestrus phase was confirmed by vaginal cytology. The vaginal smear was viewed under a light microscope at × 400 magnification. The stage of the estrous cycle was identified by the presence or absence of the nucleated epithelial cells, cornified epithelial, and leukocytes, and the cycle was divided into the four stages of proestrus, estrus, metestrus, and diestrus (25).

### Histological evaluation

After the collection of recovered grafted ovaries (n = 20/ each), they were fixed in Bouin's solution for 8 hr, embedded in paraffin wax, serially sectioned at 5 μm, mounted on slides with 5${}^{\mathrm{th}}$ intervals, and stained using the H&E method. The collected tissue sections with the mentioned interval were observed and analyzed under a microscope one by one. The morphology of tissue sections was studied under a light microscope for determining the normal and degenerated follicles at different developmental stages. The ovarian follicles were classified as primordial (oocytes surrounded by a single layer of squamous pregranulosa cells), primary (surrounded by a single layer of granulosa cells which were cuboidal), preantral (those that had more layers of cuboidal granulosa cells surrounding the oocyte, and also a clear antrum cavity), and antral follicles with the antrum cavity. The corpus luteum were defined mostly by two steroidogenic cells that rose from the cells surrounding the ovarian follicle including: thecal-lutein cells and granulosa-lutein cells. These cells have polyhedral eosinophilic cells.

To avoid counting follicles more than once, only follicles in the sections with visible oocyte nuclei were counted. Another set of tissue sections were put on coated slides and used for immunohistochemical study.

### Immunohistochemical analysis for BAX and VEGF

Immunohistochemistry was performed as described earlier (18, 26). The tissue sections (n = 10) from the experimental group and adult mouse control groups (n = 6) were deparaffinized and rehydrated in descending ethanol solutions and finally washed in phosphate buffer saline (PBS). Antigen retrieval was performed by boiling the tissue slides in 10-mM citrate buffer (10 mM, pH = 6) in a microwave oven (10 min at 700 W); they were cooled at room temperature and washed in PBS. The sections were immersed in triton × 100 (0.3% for 30 min), washed in PBS, blocked with goat serum (30 min), and incubated overnight at 4°C in a humid chamber with two antibodies separately. The primary antibodies included anti-BAX polyclonal antibody (Elabscience Biotechnology Co., Wuhan, China, 1: 100) and anti-VEGF polyclonal antibody (1: 100, Elabscience Biotechnology Co., Wuhan, China). After washing the tissue sections, they were incubated with a polyclonal goat anti-rabbit antibody (Elabscience Biotechnology Co., Wuhan, China, 1: 20) which was conjugated with FITC for 30 min. Some of the tissue sections were counterstained for the nucleus by acridine orange. Then the tissue slides were evaluated under a fluorescent microscope at × 400 magnification.

The VEGF-positive reaction among studied groups showed a similar pattern, thus it was evaluated quantitatively. Otherwise, the photos of each tissue section for BAX immunohistochemistry were prepared and imported into Image J software (National Institutes of Health, Bethesda). Then the mean number of BAX-positive cells was counted in 1,000 µ${}^{2}$ surface area in each group.

### Ethical considerations

This study was approved by the Ethics Committee for Animal Research of the Tarbiat Modares University (Ref No: IR.TMU.REC.1395.530). It does not violate the ethics working with laboratory animals.

### Statistical analysis

Data were collected and analyzed by the Statistical Package for the Social Sciences, version 17.0, SPSS (Inc, Chicago, Illinois, USA). Values are presented as Mean ± SD. One-way ANOVA and post-hoc Tukey's tests were used to compare differences in follicular count and number of apoptotic cells. P < 0.05 was considered as statistically significant.

## 3. Results

### Vaginal cytology

The mouse vaginal smear was performed at the proestrus stage bypassing 5 wk from transplantation in grafted mice. At this stage, the nucleated epithelial cells were prominent. The estrus cycles of mice were started in 60 and 70% of uni- and bilaterally ovariectomized mice after transplantation, respectively.

### Histological evaluation

**Figure 1:**
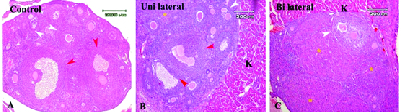
Light microscopic observation of mouse ovaries in control and experimental groups that were stained with H&E. (A) Control group, (B) Transplanted and recovered ovaries under kidney capsule in unilaterally ovariectomized group, and (C) Transplanted and recovered ovaries under kidney capsule in bilaterally ovariectomized group, Antral follicle: Red arrowheads, Preantral follicle: White arrowheads, Corpus luteum: Yellow*, K: Kidney tissue (Bar = 200 μm).

The morphological observations of grafted ovaries under the kidney capsule in uni- and bilaterally ovariectomized groups and the intact control group that was performed using the H&E staining are shown in Figures 1A-1C. The light microscopic observations of ovarian sections showed normal structure of follicles at different developmental stages such as the primordial, primary, preantral, antral follicles, and corpus luteum. As these representative figures demonstrated, the corpus luteum was prominent in the bilateral group in comparison to the other two groups.

### The percentage of normal follicles

Table I summarizes the percentages of normal follicles at different developmental stages in all studied groups. There was no significant difference among them.

Among the normal follicles counted in each group (Table I), the lowest percentages of primordial and primary follicles were seen in the bilaterally ovariectomized group compared to the other two groups (p = 0.002), while the highest percentages of primordial follicles were observed in the control group (p = 0.001).

In both ovariectomized groups, the percentages of preantral and antral follicles were higher than the intact control group (p = 0.002), while between the two transplanted groups, the bilaterally ovariectomized group had a higher percentage (p = 0.003). Moreover, the percentage of corpus luteum in the control group was significantly lower in comparison with the two ovariectomized groups (p = 0.049). Also, the highest percentage of corpus luteum was seen in the bilaterally ovariectomized group (p = 0.001).

### Immunohistochemistry for BAX 

**Figure 2:**
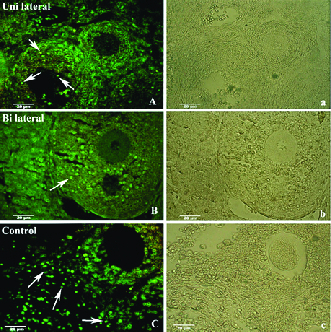
Photomicrographs of tissue sections of grafted mouse ovaries immunostained for BAX antibody under a fluorescent microscope. (A & a) Bilaterally ovariectomized groups, (B & b) Unilaterally ovariectomized group, and (C & c) Positive control, green color shows the positive cell reaction for BAX antibody (white arrow) (Bar = 20 μm).

**Figure 3:**
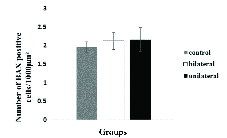
The comparison of BAX-positive cells within the tissue sections of ovariectomized and control groups. One-way ANOVA and post-hoc Tukey's tests were used to compare the differences in the number of apoptotic cells. There was no significant difference between these groups.

Figure 2A-2C show the representative photomicrographs of immunostained tissue sections in both recovered-transplanted ovaries and intact control ovaries. The BAX-positive cells were seen to have green color in preantral and antral follicles, and within the stromal tissue (Figure 2, white arrow). Moreover, small follicles indicated no BAX-positive cells in all groups. The number of BAX-positive cells within the ovaries sections in 8-wk-old mice as control and the uni- and bilateral ovariectomized mice were 1.95 ± 0.14/1000 µ${}^{2}$, 2.12 ± 0.22/1000 µ${}^{2}$, and 2.16 ± 0.32/1000 µ${}^{2}$, respectively. No significant difference was observed between these groups (Figure 3).

### Immunohistochemistry for VEGF

**Figure 4:**
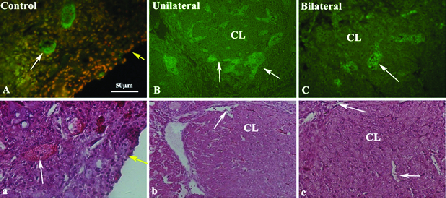
Representative micrographs of transplanted mouse ovarian sections immunostained for VEGF protein are presented in parts A-C. The H&E staining of the same groups are shown in the second row. A and a: Control group, B and b: Unilaterally ovariectomized mice, and C and c: Bilaterally ovariectomized mice. In part A, the tissue sections were also stained for the nucleus by acridine orange and the green color shows the positive cell reaction (white arrow) for the VEGF antibody in several blood vessels (Bar = 50 μm). Yellow arrows show the epithelium of the ovary, CL: Corpus luteum.

The immunostained tissue sections of recovered ovaries in both uni- and bilateral ovariectomized mice are presented in Figure 4A-4C. As shown in these figures, several blood vessels with different sizes indicated the positive reaction for VEGF. These reactions were prominent in the corpus luteum and theca layer of large follicles (Figure 4A-4C: White arrows).

**Table 1:** The mean percentage of normal follicles at different developmental stages in all studied groups


**Group**	**Total follicle**	**No. normal follicles**	**No. primordial follicles **	**No. primary follicles **	**No. preantral follicles **	**No. antral follicles **	**No. corpus luteum **
**Control (n = 6)**	509	508 (99.74 ± 0.09)	192 (37.79 ± 1.18)	97 (19.09 ± 0.88)	124 (24.40 ± 1.01)	83 (16.33 ± 0.68)	12 (2.36 ± 0.37)
**A (n = 20)**	432	430 (99.53 ± 0.04)	94 (21.86 ± 1.06)${}^{a}$	77 (17.90 ± 1.12)${}^{a}$	128 (29.76 ± 0.67)${}^{a}$	110 (25.58 ± 1.27)${}^{a}$	21 (4.88 ± 0.39)${}^{a}$
**B (n = 20)**	457	455 (99.56 ± 0.03)	44 (9.67 ± 1.12)${}^{ab}$	55 (12.08 ± 1.16)${}^{ab}$	165 (36.26 ± 1.28)${}^{ab}$	143 (31.42 ± 0.68)${}^{ab}$	48 (10.54 ± 3.88)${}^{ab}$
Data presented as n (Mean ± SD).One-way ANOVA and post-hoc Tukey's tests were used to compare the differences in the follicular count. The percentage of follicles at different developmental stages in all studied groups was calculated according to the normal follicles. Group A: Ovariectomized group, Group B: Bilateral ovariectomized group, ${}^{a}$Significant differences with the control group (p < 0.049), ${}^{b}$Significant with a unilaterally ovariectomized group (p < 0.049)							

## 4. Discussion

Five weeks after the transplantation, the morphology of transplanted ovaries was well preserved and the percentage of normal follicles was not significantly different, compared to the intact control ovaries. This result may be related to the site of the transplantation, due to the reason that the kidney capsule has a rich vascular supply. Similarly, it was indicated that the transplantation of ovaries into the kidney capsule is a promising site for ovarian tissue in mice (12). In correlation with our morphological observations in other parts of this study, no significant increase was observed in the number of BAX-positive cells in both transplanted groups, compared to the intact control group. Moreover, the presence of BAX-positive cells in large follicles has been considered for the regulation of atresia within these follicles by program cell death.

As the literature showed, the main challenge in the transplanted tissue is the ischemia in regard to the hypoxic condition that could affect the quality of transplanted tissue, resulting in a reduction of the follicular development. We suggest that the sub-capsule of the kidney could be considered as a suitable site for transplantation of ovaries, due to its high blood supply. On the other hand, cell death and follicular degeneration mainly take place in the short term after transplantation and before revascularization of grafted tissue.

An increase in the cell death has been reported to be observed shortly after the transplantation (19). However, in the present study, we evaluated the incidence of apoptosis bypassing 5 wk from transplantation; thus, the apoptotic cells may be removed from tissue at this time.

From the other point of view, the results of the present study showed that the speed of follicular development in both grafted ovaries was significantly faster than the intact control for one time (the percentage of preantral and antral follicles and corpus luteum were significantly higher in both transplanted groups). This phenomenon demonstrated the rapid development and early discharge of ovarian reserve in both transplanted groups, which could affect the longevity of transplanted tissue. These effects may be related to an imbalance between regulatory factors that control the follicular development in ovariectomized mice. Similarly, the same observation has been also observed in a variety of species, which indicated that the removal of one ovary increased the rate of follicular development in the remaining ovary (21-23). Moreover, the percentages of antral follicles in combination with corpus luteum in the bilaterally ovariectomized group were significantly higher than the unilaterally ovariectomized group. It was shown that the follicular development in the unilaterally ovariectomized group is closer to the intact group than the bilaterally ovariectomized group. This difference may be related to regulatory factors that were locally secreted by the remaining ovary in the unilaterally ovariectomized group (5). Although in our study, the hormonal profiles of the mice were not evaluated, previous studies have shown that the concentrations of follicle-stimulating hormone (FSH) and luteinizing hormone (LH) were significantly increased in the sera of bilaterally ovariectomized in comparison with the unilaterally ovariectomized animal of the same age (22). Therefore, it was suggested that the high level of FSH in bilaterally ovariectomized recipients could be correlated with the premature follicular discharge in this group.

In unilaterally ovariectomized mice, the remaining ovary could produce enough estradiol and inhibition to sustain negative feedback on the hypothalamus-pituitary ovary axis, resulting in lower FSH level, compared to bilaterally ovariectomized group (27). While in the latter group, an immediate lack of negative feedback after grafting could facilitate the production of gonadotropin, which releases GnRH, FSH, and LH hormones leading to improved revascularization and growth of follicles in grafted ovaries (15, 27-29).

From a different perspective, the angiogenesis in the grafted tissue could improve follicular development. Our observation demonstrated the VEGF-positive cells within the recovered ovaries in both ovariectomized groups. As reported in the literature, VEGF as an important factor has the ability to induce angiogenesis in grafted tissue (9-11). Also, angiogenesis is critical for the formation of the theca layer during follicular growth and corpus luteum development (11).

## 5. Conclusion

Rapid follicular development and early discharge of ovarian reserve were seen in both transplanted groups; however, it was prominent in the bilaterally ovariectomized group. Also, the incidence of apoptosis and the expression of VEGF as an angiogenic factor did not differ in the transplanted ovaries of uni- and bilaterally ovariectomized mice. Thus, unilateral ovariectomy has less side effects on the ovarian reserve in comparison with the bilaterally ovariectomy.

##  Conflict of Interest

None declared.
